# Five-Year Follow-Up of Photobiomodulation in Parkinson’s Disease: A Case Series Exploring Clinical Stability and Microbiome Modulation

**DOI:** 10.3390/jcm15010368

**Published:** 2026-01-04

**Authors:** Brian Bicknell, Ann Liebert, Craig McLachlan, Hosen Kiat

**Affiliations:** 1 Brain and Mind Centre, Sydney University, Camperdown, NSW 2050, Australia; 2 Kolling Institute, Sydney University, St Leonards, NSW 2064, Australia; ann.liebert@sydney.edu.au; 3 Centre for Healthy Futures, Torrens University, Sydney, NSW 2000, Australia; craig.mclachlan@torrens.edu.au (C.M.); hosen.kiat@mq.edu.au (H.K.); 4 Department of Medicine, Faculty of Medicine and Health Sciences, Macquarie University, Sydney, NSW 2109, Australia; 5 Department of Medicine, ANU College of Health and Medicine, Australian National University, Canberra, ACT 2601, Australia

**Keywords:** Parkinson’s disease, microbiome, photobiomodulation, short-chain fatty acids, lipopolysaccharide, hydrogen sulphide

## Abstract

**Background**: Parkinson’s disease (PD) involves progressive neurodegeneration with clinical or subclinical disturbance of the gut–brain axis, including altered gastrointestinal motility and enteric nervous system involvement. Clinical studies have reported gut microbiome alterations in PD, with shifts in taxa associated with inflammatory signalling and short-chain fatty acid (SCFA) metabolism. Photobiomodulation (PBM), a non-invasive light therapy, has been investigated as a potential adjunctive treatment for PD, with proposed effects on neural, metabolic, and immune pathways. We previously reported the five-year clinical outcomes in a PBM-treated Parkinson’s disease case series. Here we report the five-year gut microbiome outcomes based on longitudinal samples collected from the same participants. This was an exploratory, open-label longitudinal study without a control group. **Objective**: Our objective was to assess whether long-term PBM was associated with changes in gut microbiome diversity and composition in the same Parkinson’s disease cohort as previously assessed for changes in Parkinson’s symptoms. **Methods**: Six participants from the earlier PBM proof-of-concept study who had been diagnosed with idiopathic PD and who had continued treatment (transcranial light emitting diode [LED] plus abdominal and neck laser) for five years had their faecal samples analysed by 16S rDNA sequencing to assess microbiome diversity and taxonomic composition. **Results**: Microbiome analysis revealed significantly reduced evenness (α-diversity) and significant shifts in β-diversity over five years, as assessed by Permutational Multivariate Analysis of Variance (PERMANOVA). At the phylum level, Pseudomonadota and Methanobacteriota decreased in four of the six participants. Both of these phyla are often increased in the Parkinson’s microbiome compared with the microbiomes of healthy controls. Family-level changes included increased acetate-producing Bifidobacteriaceae (five of the six participants); decreased pro-inflammatory, lipopolysaccharide (LPS)-producing Enterobacteriaceae (two of the three participants who have this bacterial family present); and decreased LPS- and H_2_S-producing Desulfovibrionaceae (five of six). At the genus level, *Faecalibacterium*, a key butyrate producer, increased in four of the six participants, potentially leading to more SCFA availability, although other SCFA-producing bacteria were decreased. This was accompanied by reductions in pro-inflammatory LPS and H_2_S-producing genera that are often increased in the Parkinson’s microbiome. **Conclusions**: This five-year case series represents the longest follow-up of microbiome changes in Parkinson’s disease, although the interpretation of results is limited by very small numbers, the lack of a control group, and the inability to control for lifestyle influences such as dietary changes. While causal relationships cannot be inferred, the parallel changes in improvements in mobility and non-motor Parkinson’s symptoms observed in this cohort, raises the hypothesis that PBM may interact with the gut–brain axis via the microbiome. Controlled studies incorporating functional multi-omics are needed to clarify potential mechanistic links between microbial function, host metabolism, and clinical outcomes.

## 1. Introduction

Parkinson’s disease (PD) is a progressive neurodegenerative disorder characterised by α-synuclein aggregation, dopaminergic neuronal loss in the substantia nigra, and attendant motor and non-motor symptoms [1]. It is increasingly recognised as a multisystem disorder, involving both central neurological changes and peripheral disturbances of the gut–brain axis. It has recently been hypothesised that the heterogeneous nature of Parkinson’s symptoms might be explained by PD being acquired as brain-first (central nervous system [CNS]) or body-first (peripheral), depending on the origin of the α-synuclein pathology [2]. There is ample evidence from animal models that α-synuclein aggregation can originate in the enteric nervous system and then spread to the CNS [3,4]. Converging evidence has highlighted the role of gut–brain axis dysfunction in PD pathophysiology [5,6,7], with dysbiosis in the gut microbiome being a common feature of idiopathic PD [8,9,10,11] with microbiome differences in the body-first and brain-first subtypes [12]. Gastrointestinal symptoms, including microbiome disruption characterised by loss of short-chain fatty acid (SCFA) producing taxa and increases in pro-inflammatory bacteria, often precede motor manifestations by years, suggesting early involvement of the gut and its microbiome [13]. Microbiome changes have been linked to impaired intestinal integrity, heightened inflammation, and increased neural vulnerability [10,14,15], as well as PD progression [9,16,17,18].

Current microbiome-targeted interventions for PD, including dietary modification, prebiotics, probiotics, and faecal microbiota transplantation (FMT), show promise but with inconsistent clinical benefits, highlighting the need for novel approaches to modulate the microbiome-gut–brain axis.

Photobiomodulation (PBM) is a non-invasive therapy using non-thermal red to near-infrared light [19,20], and has shown neuroprotective and immunomodulatory effects in preclinical PD models [21,22], with emerging evidence from clinical trials [23,24,25,26]. Early reports also indicate that PBM might modulate the gut microbiome in animal models [27] and in small human studies [28,29], including increases in beneficial SCFA producers and reductions in pro-inflammatory bacteria. We have previously described sustained clinical stability in a small cohort of study participants who have used long-term PBM therapy [24]. The present study builds directly on these clinical observations by examining the corresponding five-year gut microbiome profiles in the same participants, providing an exploratory insight into the effect of prolonged PBM on longitudinal changes in the microbial composition of the gut while acknowledging that other factors, such as dietary changes, aging, and medications, may also influence microbiome changes.

## 2. Methods

### 2.1. Study Design and Participants

This was an open-label, longitudinal follow-up study of participants from a previously reported PBM proof-of-concept study of PD [24,26]. Six participants from an original proof-of-concept study, with clinically diagnosed idiopathic PD, continued PBM treatment for five years (Table 1). The remaining six participants from the original study had either been diagnosed with an alternative disease (multisystem atrophy, two participants) or had discontinued PBM treatment due to their partner passing away and relocation to an aged care facility (one participant); recovery from cancer therapy (one participant); or declining to continue PBM treatment (two participants). All continuing participants had participated in earlier PBM studies conducted under approved clinical research protocols and provided renewed informed consent. All indicated that they had continued PBM therapy with varying degrees of consistency for five years [24].

### 2.2. Intervention

Participants self-administered at-home PBM three times weekly using a combination of transcranial, cervical, and abdominal irradiation, as previously described [26]. The abdomen and neck were irradiated using a near-infrared 904 nm Class 1 laser and one of three light emitting diode (LED) devices on the head (Supplementary Table S1). Treatment protocols were based on previously published PBM parameters shown to modulate motor and cognitive outcomes in PD and were maintained with minimal adjustment throughout the five-year period. There were a number of confounding factors that could have contributed to microbiome changes over time, such as dietary changes, aging, medications, and changes in exercise patterns. These were not directly monitored but self-reported by participants at the 5-year assessment.

### 2.3. Sample Collection, DNA Extraction, and 16S rRNA Gene Sequencing

Stool samples were collected from each participant at baseline and after five years of continuous PBM. Samples were immediately frozen at −80 °C until analysis. Total bacterial DNA was extracted using the Qiagen PowerSoil DNA Isolation Kit following the manufacturer’s instructions, with modifications to optimise yield from low-biomass samples. DNA quality and concentration were verified by NanoDrop spectrophotometry and agarose gel electrophoresis.

All sequencing and bioinformatics analyses were performed with participant identity masked. 16S rRNA gene sequencing was chosen to maintain methodological consistency with prior analyses and as a cost-effective method for community-level profiling. The V3–V4 regions of the bacterial 16S rRNA gene were amplified using universal primers and sequenced on an Illumina (San Diego, CA, USA) MiSeq platform (2 × 300 bp paired-end reads) in two separate sequencing runs (baseline and 5 years). Raw reads were quality-filtered, denoised, and clustered into amplicon sequence variants (ASVs) using the QIIME 2 pipeline (v2024.4) with DADA2 [30]. All samples were retained after denoising. The total number of sequencing reads was 933,105, with a mean sequencing depth of 70,758. Sampling depth (33,000) was set to the shallowest sample (A2 baseline), ensuring that Shannon alpha-rarefaction had plateaued for all samples. Negative extraction and sequencing controls were included, and sequence quality metrics were assessed prior to analysis. No contaminant signatures were identified in the controls or samples; thus, decontamination tools were not applied. Taxonomy was assigned against the Greengenes2 database [31]. Alpha (α-) diversity metrics (Observed Features, Shannon index, Faith’s Phylogenetic Diversity, and Pielou Evenness Index) were computed with QIIME 2 using the Kruskal–Wallis Test. Beta (β-) diversity was assessed using permutational multivariate analysis of variance (PERMANOVA), with 999 iterations for unweighted and weighted UniFrac distances visualised by principal coordinate analysis using the EMPeror plugin [32]. Changes in genus abundance between groups were tested with the Analysis of Compositions of Microbiomes (ANCOM) plugin [33] after centred log-ratio transformation. Relative abundance was determined at the phylum, family, and genus levels. At the phylum level, the various Bacillota phyla were combined to allow comparison with earlier studies. Taxa changes were exploratory, using a 2-fold difference in relative abundance as the threshold for change. Taxonomic changes were interpreted with reference to published PD microbiome signatures [9,10,11,16,34,35,36,37,38,39,40,41,42,43,44,45], with particular attention to taxa associated with SCFA production and with inflammatory potential.

### 2.4. Ethical Approval

All procedures complied with the Declaration of Helsinki and were approved by the Griffith University Human Research Ethics Committee (approval code 2018/16, approved 3 February 2018) with an extension until 24 April 2024. This study was registered with the Australian New Zealand Clinical Trials Registry (ANZCTR—a primary registry in the WHO International Clinical Trial Registry Platform), registration number ACTRN12618000038291p, registered on 12 January 2018. Written informed consent was obtained from all participants.

## 3. Results

Participants did not report any adverse events due to the PBM treatment. The participants reported that they had no major dietary changes during the 5-year PBM intervention, although one indicated that their diet may have been “less healthy” compared with the first year and one that they had reduced their carbohydrate intake at 2 years in order to lose weight (Table 1). The participants had been diagnosed with PD between 2 and 7 years before beginning this study in 2019, and five of the six participants showed no decline in Movement Disorder Society Unified Parkinson’s disease Rating Scale (MDS-UPDRS-III [motor examination]) scores over the 5-year intervention period (Table 1). All participants were using dopamine replacement therapy.

### 3.1. Microbiome Diversity

**Figure 1:**
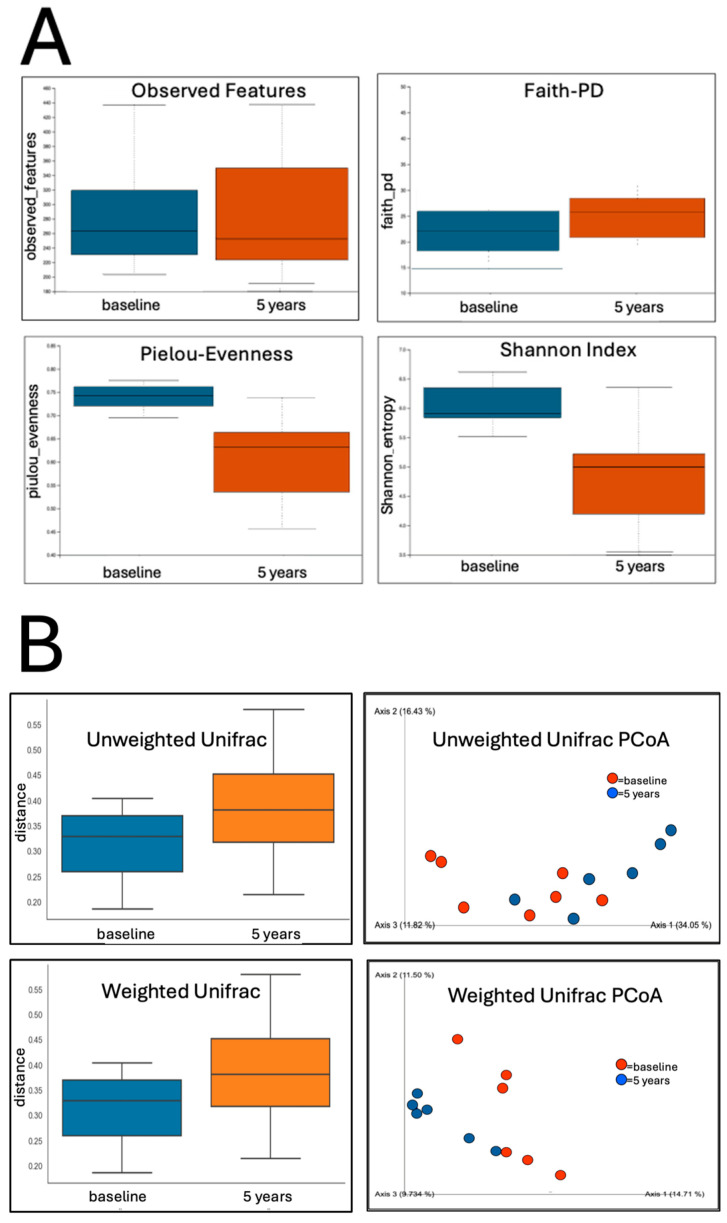
Diversity analysis of gut microbiome samples before PBM treatment (baseline) and after 5 years of PBM treatment. (**A**) The α-diversity showing no significant differences in richness indices (Observed Features and Faith’s PD), but significant differences in the Pielou’s Evenness Index (Kruskal–Wallis test, q = 0.016) and the Shannon index (Kruskal–Wallis test, q = 0.025). (**B**) The β-diversity, showing significant differences between baseline and 5 years for unweighted UniFrac (PERMANOVA, q = 0.003) and weighted UniFrac (PERMANOVA, q = 0.036), as well as PCoA plots. Blue = baseline; Orange = 5 years.

There was no significant change in the α-diversity measures of richness as determined by the Observed Features measure or Faith’s Phylogenetic Diversity. However, Pielou’s Evenness Index was significantly reduced (Kruskal–Wallis test, *q* = 0.016), as was the Shannon Index (Kruskal–Wallis test, *q* = 0.025) (Figure 1A). There was also a significant shift in β-diversity between baseline and 5 years for both unweighted and weighted UniFrac metrics (PERMANOVA, q = 0.003 and q = 0.036, respectively) (Figure 1B).

### 3.2. Taxonomic Changes

Changes in microbiome composition at the phylum, family, and genus taxonomic levels are shown in Supplementary Table S2. Taxonomic changes and their functional interpretations are summarised in Table 2.

**Figure 2:**
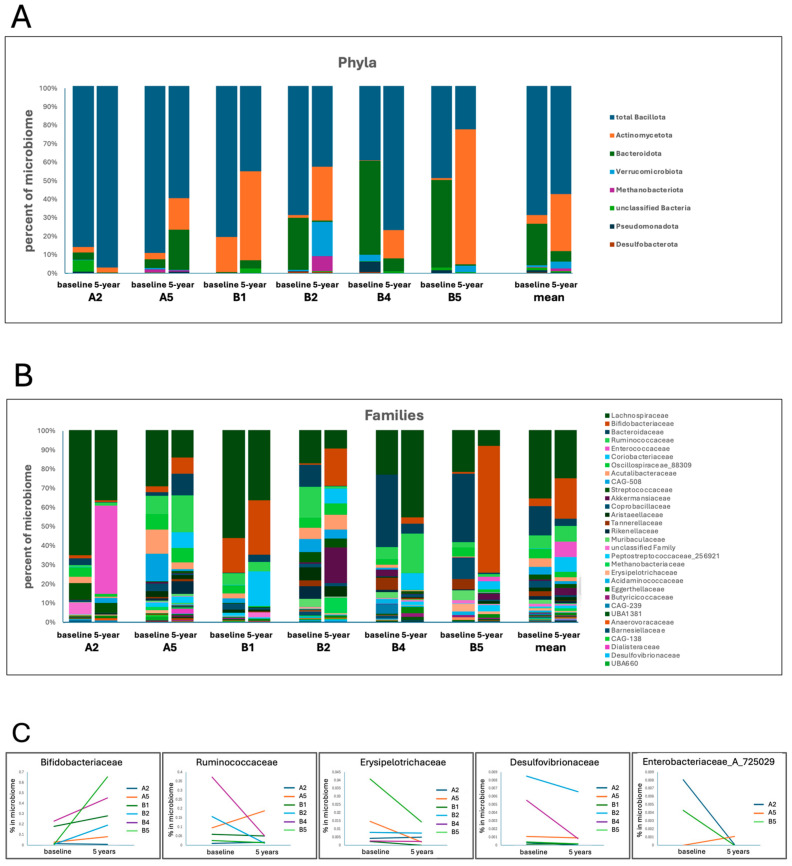
Taxonomic changes in the gut microbiome from before PBM treatment (baseline) to after 5 years of PBM treatment at the phylum and family taxonomic levels. (**A**) Composition of phyla for each participant and mean composition before PBM treatment and after 5 years of treatment. (**B**) Composition of families for each participant and mean composition before PBM treatment and after 5 years of treatment. (**C**) Selected family changes from baseline to after 5 years of PBM treatment.

Phylum-level changes (Figure 2A) in the eight most common phyla showed increases in Actinomycetota in all participants (six of six) and decreases in unclassified bacteria (five of six), Bacillota (four of six), Bacteroidota (four of six), and Pseudomonadota (four of six). 

Family-level changes (Figure 2B) in the 45 most common families showed increases in Bifidobacteriaceae in five of the six participants and Ruminococcaceae in half of the participants (Figure 2C). Enterobacteriaceae, Erysipelotrichaceae, and Desulfovibrionaceae were mostly decreased in 5-year microbiomes (Figure 2C).

**Figure 3:**
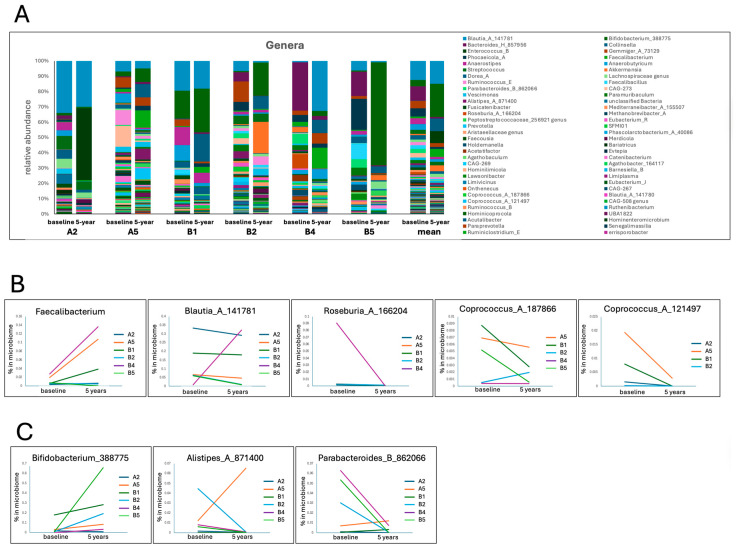

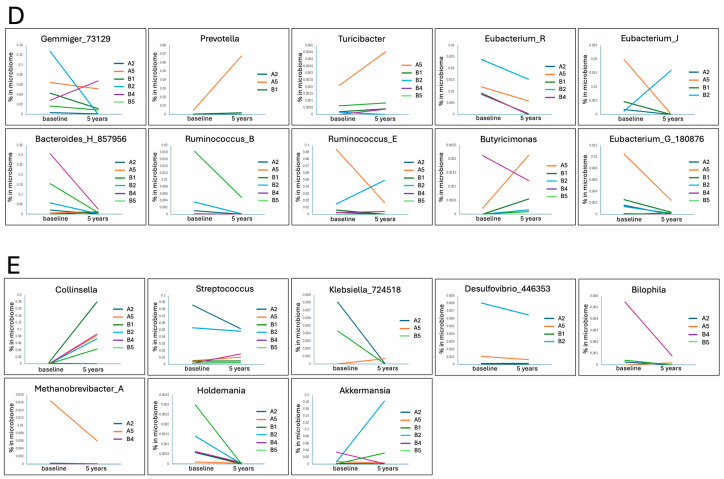
Taxonomic changes in the gut microbiome from before PBM treatment (baseline) to after 5 years of PBM treatment at the genus level. (**A**) Composition of genera for each participant and mean composition before PBM treatment and after 5 years of treatment. (**B**) Selected genus changes from baseline to after 5 years of PBM for genera reported in the literature as SCFA-producing and depleted in the PD microbiome. (**C**) Selected genus changes from baseline to after 5 years of PBM for genera reported in the literature as SCFA-producing and enriched in the PD microbiome. (**D**) Selected genus changes from baseline to after 5 years of PBM for genera reported in the literature as SCFA-producing and either enriched or depleted in the PD microbiome. (**E**) Selected genus changes from baseline to after 5 years of PBM for genera reported in the literature as pathobionts and/or pro-inflammatory.

Genus-level changes for the 122 most common genera showed that SCFA-producing bacteria both increased and decreased over 5 years. *Faecalibacterium* was increased in four of the six participants in the current study (Figure 3B): in one participant (B4), increased to 13.6% of the microbiome. Other SCFA-producing bacteria, *Roseburia*_A_166204 and *Roseburia*_*C*, showed decreases in five of five and four of four participants, respectively, from low proportions (<1%) in most participants. *Anaerostipes* decreased in two of the six participants, with the remaining four showing no change. *Blautia_A_141781* was overall the most common genus, representing up to 32.2% of the microbiome. *Blautia* decreased in two participants with lower proportions of the genus in their microbiomes (6.07% and 0.80%). Other prominent SCFA producers that have been reported as depleted in PD microbiomes (such as *Butyrivibrio* and *Butyribacterium*) were either detected at very low levels (in one participant only) or not detected, suggesting that these may have been substantially reduced or eliminated during the years of dysbiosis. Other SCFA-producing genera have been reported as being increased in PD microbiomes (Table 2), such as *Bifidobacterium,* which increased in four of the six participants, while *Alistipes* and *Parabacteroides* decreased in the majority of participants (Figure 3C). SCFA-producing genera that have been reported as increased in PD in some studies and decreased in other studies (Table 2) also showed either increases in the majority of participants (*Butyricimonas, Prevotella, Turicibacter*) or decreases (*Bacteroides* and the various *Eubacterium* genera) (Figure 3D).

Many pathobionts and pro-inflammatory genera that were detected in the 122 most common genera showed a decline in the majority of participants (Figure 3E), including *Bilophila* (three of four), *Desulfovibrio* (two of four, with two unchanged), *Methanobrevibacter* (two of three), and *Klebsiella* (two of three), while *Limiplasma* increased in two and decreased in two participants. *Streptococcus* increased in one participant and was unchanged in the other five. In each of these cases, the genera were at low levels (<1%) at baseline, apart from two participants with higher proportions of *Streptococcus* (8.59% and 5.32%), both of whom showed a small (less than 2-fold) decrease over 5 years. *Collinsella* is another pathobiont that is increased in the PD microbiome. It was the only genus to show a significant change (increase) over the 5 years, as detected by the ANCOM statistic, and was increased in five of the six participants to between 4% and 18% of the microbiome. Another conspicuous pathobiont change was the increase in *Enterococcus_B* in one participant (A2) from non-detected at baseline to becoming the dominant genus at 5 years (45.5% of the total microbiota).

Genera considered to be generally healthy in other contexts but that are increased in the PD microbiome include *Akkermansia, Bifidobacterium*, and *Lactobacillus* (Table 2). *Akkermansia* decreased in four of the six participants in the current study (Figure 3E), although one participant showed an increase in *Akkermansia* to 18% of the microbiome. The proportion of *Lactobacillus* in the samples was below the threshold for inclusion in this analysis.

There are a number of genera that have been considered to be potential markers of PD progression (Table 2). Putative bacterial markers correlated with PD severity and more rapid PD progression include Desulfovibrionaceae, Erysipelotrichaceae, *Desulfovibrio*, *Bilophila*, *Limiplasma*, and *Eubacterium*, all of which, apart from *Limiplasma*, showed decreases over 5 years in the majority of participants. Putative bacterial markers inversely correlated with PD progression (*Bifidobacterium*, *Prevotella, Butyricimonas*) showed increases in the majority of participants, except for *Bacteroides*, which was decreased in the majority of participants.

## 4. Discussion

### 4.1. Longterm Clinical Stability

This study provides the longest reported follow-up of individuals with PD who have continued PBM therapy, extending the previous report of five-year clinical outcomes [24], to include parallel gut microbiome observations. In the earlier clinical analysis of this cohort, most participants who continued PBM therapy demonstrated sustained stability or improvement in mobility, balance, and non-motor features such as cognition and olfaction, with no serious adverse events reported. This stability or improvement in the MDS-UPDRS-III score can be contrasted with the expected decline of between 1.4 and 8.9 points annually in untreated or L-dopa-medicated PD patients [84].

Taken together with the changes in the microbiome, the findings suggest that long-term PBM was well-tolerated in this small cohort and was associated with sustained clinical stability alongside longitudinal changes in gut microbiome composition. While no causal inferences can be drawn, the co-occurrence of clinical stability and microbiome shifts provides a basis for further investigation in controlled studies.

### 4.2. Microbiome Shifts

Participants reported no major changes to their diets over the five years of this study, suggesting that large dietary changes were unlikely to account for the observed microbiome changes. Significant longitudinal changes were detected in both α- and β-diversity. The reduction in α-diversity evenness, reflected by lower Pielou’s Evenness and Shannon indices, indicates increasing dominance of specific microbial taxa within the microbiome community over time. While cross-sectional studies of PD have not consistently reported differences in α-diversity compared to healthy controls (HCs), several studies have described a significant increase in the α-diversity in PD cohorts compared to HCs [35,69,85,86].

Significant changes in both unweighted and weighted UniFrac indices indicate global restructuring of the microbial community. Changes in unweighted UniFrac are consistent with shifts in the presence or absence of microbial lineages, whereas changes in weighted UniFrac suggest alterations in the relative proportions of taxa present. Significant differences in β-diversity are commonly reported in cross-sectional studies [9,56], and the present findings demonstrate that comparable compositional shifts can also occur longitudinally within individuals.

To further interpret diversity shifts, an exploratory assessment of changes in taxonomic composition was undertaken. At the phylum level, reductions were observed in the relative proportion of Pseudomonadota (formally Proteobacteria), a group frequently reported as increased in PD microbiomes and with many pathobiont and pro-inflammatory members [74]. A reduction in Methanobacteriota was also observed. This is a phylum of methane-producing archaea that are also reported as increased in PD microbiomes [9]. At the family level, the relative abundance of Bifidobacteriaceae increased. Although this family includes probiotic strains, it has been reported as increased in PD [38,51]. Decreases were observed in families containing bacteria that produce LPS and H_2_S (Enterococcaceae and Desulfovibrionaceae); metabolites that have been linked to gut barrier dysfunction, inflammation, neuroinflammation, and α-synuclein aggregation [17,41,49,52,87]. Reductions were also noted in Erysipelotrichaceae, a family with strains that have been linked to gastrointestinal disruption [53] and an altered lipid metabolism [54].

Many SCFA-producing genera are reported to be decreased in the PD microbiome compared to HCs (Table 2), including the archetypal SCFA producer *Faecalibacterium,* which has been identified as one of the main producers of butyrate in the gut [88] and has consistently been reported as depleted in PD [56,57,58]. Enrichment of *Faecalibacterium, Bifidobacterium, Turicibacter, Butyricimonas*, and the Ruminococcaceae, as well as other SCFA producers, suggests increased SCFA production in the gut, although production of SCFA is strain-specific [89] and can also be affected by diet, host factors, and the gut environment [90]. The reduction of other SCFA-producing genera (*Roseburia, Coprococcus*, *Eubacterium, Bacteroides, Gemmiger*) underscores the complexity of the changes in the microbiome. The reported enrichment in the PD microbiome of taxa that are, in other contexts considered healthy (*Akkermansia*, *Bifidobacterium, Lactobacillus*) similarly suggests strain-specific and host-specific effects [38,51]. *Akkermansia,* for example, despite supporting mucin production and gut barrier health in some settings [15,81], has been linked to systemic inflammation and symptom progression in some PD studies [38,57] and was reduced in most participants in the present study.

In parallel with these changes in some putative beneficial taxa, there were decreases in *Klebsiella*, *Bilophila*, and *Desulfovibrio*, all of which are commonly enriched in PD dysbiosis [9,39,87], which suggests reduced exposure to LPS and H_2_S in the microbiomes of our participants, and may indicate some stabilisation of metabolic risk pathways. A notable exception was the significant increase in *Collinsella,* which has been implicated in increased gut permeability and pro-inflammatory signalling [35] and is reported as associated with PD dysbiosis [36,66], PD progression [35] and Lewy Body dementia [77]. Increases in this genus highlight the need for strain-level and functional profiling, as taxa may exert context-dependent effects [35,83,91,92]. The extremely high proportion of *Enterococcus_B* observed in one participant at 5 years is most likely indicative of an infection or treatment with antibiotics. *Enterococcus faecium*, a notable species in this genus, is commensal in the gut at low levels but is also known as an opportunistic pathogen with multiple antibiotic resistances and can dominate the gut microbiome in response to broad-spectrum antibiotic exposure [93].

The microbiome findings somewhat align with cross-sectional studies that have reported depletion of SCFA producers and enrichment of pro-inflammatory taxa in PD compared to HCs. Taken together, these exploratory taxonomic changes, including increases in the relative proportion of some SCFA producers and reductions in the proportion of several pathobionts, support the hypothesis that, in parallel with clinical stability, PBM might be associated with a shift towards a more metabolically supportive and less pro-inflammatory gut environment. However, alternative explanations for the changes in the microbiome in these participants (such as diet, lifestyle, natural fluctuations, etc.) cannot be excluded.

Notably, the microbiome changes observed in this study differ from the relative stability of the PD microbiome reported in other longitudinal studies [34,94]. However, the decreases in some SCFA-producing genera, including *Roseburia*, and the increases in *Bifidobacterium* and other genera that are reported as increased in PD, together with the marked increase in *Collinsella,* highlight the complexity of interpreting longitudinal microbial shifts.

### 4.3. Mechanistic Links Between PBM and the Microbiome

The mechanism by which PBM might influence the microbiome remains incompletely defined. In animal models, direct abdominal irradiation has been demonstrated in a number of studies to increase SCFA producers and reduce pro-inflammatory taxa [27,95,96,97,98,99], and direct irradiation of human faecal samples has been reported to restore cryo-damaged microbiota [100]. However, since photon penetration is limited to a few centimetres, light will not reach the interior of the gut in humans and can have no direct effect on the microbiota. Thus, interaction of PBM with the gut microbiome would be indirect. PBM has a well-known anti-inflammatory effect, directly modulating the inflammatory process by reducing pro-inflammatory cytokines (interleukin [IL]-1β, IL-6, Tumor Necrosis Factor alpha [TNF-α]) and increasing anti-inflammatory cytokines (IL-10), as well as modifying pro-inflammatory macrophages (M1) to the anti-inflammatory phenotype (M2) [101]. In addition, in animal models, PBM has been shown to modulate extracellular signal-regulated kinase (ERK), influencing the mitogen-activated protein kinase (MAPK) pathways [102,103], which in turn influences the gut-associated lymphoid tissue (GALT) and reduces local abdominal and mucosal inflammation [104,105,106]. Evidence suggests that reducing inflammation has the effect of enhancing gut barrier integrity [107]. PBM also reduces oxidative stress [104], potentially reversing damage to the gut colonic epithelial cells. Improved gut barrier integrity and reduced inflammation create an environment that favours beneficial taxa such as *Faecalibacterium* while suppressing LPS- and H_2_S-producing bacteria. We could hypothesise that this convergence of reduced systemic inflammation, improved barrier function, and ecological shifts may go some way to explain both the observed stability in clinical outcomes and changes in microbial composition in the gut.

### 4.4. Comparison with Other Microbiome-Targeted Interventions

Other microbiome-targeting strategies in PD, such as diet, prebiotics, probiotics, synbiotics, exercise, and FMT, have produced mixed results. Mediterranean and fibre-rich diets appear protective epidemiologically but show inconsistent benefits after PD onset [108,109]. Prebiotics, probiotics, and synbiotics improve dysbiosis and reduce inflammation in animal models [110], but human trials can show selective microbial changes without a lasting clinical impact [111,112,113]. While moderate exercise is known to improve the microbiome in healthy individuals [114], evidence for this effect in PD is lacking. FMT, while highly effective in animal models, has yielded inconsistent clinical results [115,116], with a recent clinical study showing no consistent improvement in motor or non-motor symptoms with 6 months of FMT [117]. Most interventions, with the possible exception of FMT, are comparatively simple to administer. Compared with these interventions, PBM offers several advantages: it is non-invasive and well-tolerated, has a well-documented safety profile, and has shown lasting improvements in both motor and non-motor symptoms of PD in a number of studies [23,24,25,26].

In an aging non-PD population, the microbiome would be expected to deteriorate, with reduced SCFA-producing bacteria and increased pro-inflammatory bacteria [118,119]. In the PD microbiome, we would not expect an improvement in the gut microbiome over time [120]. The observed parallel clinical improvement in the symptoms of PD and the changes in the microbiome suggest the hypothesis that there may be a link between modulation of the microbiome with PBM, the gut–brain axis, and symptomatic improvement.

## 5. Limitations

Our study findings should be interpreted with great caution. The very small sample size limits statistical power and generalisability, and individual variation in temporal changes in the microbiome highlights the complexity of host–microbe interactions in PD and limits the extent to which the results can be generalised. Confounding factors that might have contributed to microbiome changes were not closely monitored. Dietary changes, although minimal according to participant responses, might have influenced the microbiome changes. Changes in medication, the effect of aging, and changes in exercise and activity level (made possible by improved Parkinson’s symptoms) might also have affected the results. It is also possible that the PBM protocol was not strictly adhered to by the participants, and in fact all participants acknowledged some flexibility in following the treatment regimen but also commented that symptom changes when PBM sessions were missed had prompted renewed adherence to the regimen.

Sequencing was restricted to 16S rDNA rather than metagenomics. 16S rDNA sequencing is inaccurate in species-level identification and cannot track functional changes in the microbiome. Future studies should use whole-metagenome sequencing. In addition, sequencing of samples was undertaken on two occasions (July 2019 for baseline samples and May 2024 for 5-year samples), which reduces the confidence in the significance of α- and β-diversity changes. While the conditions to use PERMANOVA were met, the number of paired samples (six) was towards the lower limit for this statistic. Interpretation of the microbiome changes is also limited by the inherent temporal variability iof the microbiome and the variability of microbiota in our participants and in PD generally.

No analysis of metabolites such as SCFA, H_2_S, or inflammatory markers was undertaken, which would have added to this study. In addition, this study would have benefitted from measures of gut integrity, such as zonulin and calprotectin tests. The absence of a control group also leaves placebo and lifestyle effects unresolved. Although the clinical stability over five years is encouraging, a direct causal link to PBM cannot yet be confirmed.

### Future Directions

Future studies should include larger, well-controlled randomised trials with extended follow-up, integrating multi-omics approaches such as metagenomics, metabolomics, and immunophenotyping to link microbial function with host responses. Strain-level analysis of *Akkermansia, Bifidobacterium,* and *Collinsella* is particularly important, given their context-dependent roles. Mechanistic work on PBM’s interaction with GALT and systemic inflammation and its combination with dietary fibre or probiotics may reveal additive or synergistic benefits.

## 6. Conclusions

This five-year case series is the longest to date examining PBM outcomes in PD. While exploratory, the results suggest that PBM not only stabilises motor signs, cognitive function, sense of smell, and other non-motor symptoms of PD over an extended period but may also influence the microbiome structure. We might hypothesise that PBM could promote an overall improved microbiome composition and that, while not achieving a completely healthy (eubiosis) microbiome, the resultant microbiome changes might be less inflammatory and more enriched in SCFAs, and the parallel improvement in clinical outcomes might be facilitated via the microbiome-gut–brain axis. Larger, rigorously controlled trials incorporating functional multi-omics are needed to clarify the mechanistic links between microbial activity, host metabolism, and disease modification.

## Figures and Tables

**Table 1:** Demographic characteristics of participants.

		Participants					
		A2	A5	B1	B2	B4	B5
Sex		F	M	F	F	M	F
Age	Baseline	79	72	58	77	75	67
	5 years	84	77	63	82	80	72
Years since diagnosis at baseline		1	not reported	3	7	2	7
Hoehn and Yahr stage	Baseline	2	2	2	3	1	2
	5 years	3	2	2	3	2	2
Affected side		L	L	L	L	L	R
Medications		Madopar bd HBS nocte	Sinemet 7 × d Sifrol mane	Kinson 7 × d	Kinson QID	Madopar bd	Stalevo QID
Daily L-dopa		600 mg	700 mg	170 mg	400 mg	600 mg	800 mg
MDS-UPDRS-III SCORE	Baseline	20	15	23	54	18	20
	5 years	24	15	21	23	12	19
Falls in 5 years		0	0	0	0	0	0
Change in sense of smell		Improvement from hyposmia	Unchanged	Slow improvement	Substantial improvement from >5 years of anosmia	Improvement from anosmia	Slowly deteriorating
Major dietary changes over 5 yrs		No	No	? less healthy after year 2	No	No	Reduced carbohydrates 2 years into study
Exercise		Bike 20 min/day	Unanswered	Bike 30–40 km/week	Gardening + incidental (stairs)	Walking 5–6000 steps/day	PD-specific exercises 1× per week
Helmet used		SYMBYX	VIELIGHT	SYMBYX	WELL RED	VIELIGHT	SYMBYX

F = female; M = male; L = left side; R = right side; bd = twice daily; QID = 4 times daily; nocte = at night; 7 × d = 7 times per day; mane = in the morning; ? = possibly.

**Table 2:** Phylum-, family-, and genus-level microbiome changes after 5 years of PBM in a small PD cohort (n = 6). Taxa changes are presented primarily as exploratory, using a 2-fold difference in relative abundance as the threshold for change. Statistical inference using ANCOM identified a significant increase in *Collinsella*.

Taxa	Functional Relevance	Change in PD vs. HCs	Change over 5 Years				Mean % in
			Incr.	Decr.	nc	nd	Microbiome
Phylum							
Bacillota	Contains many SCFA producers	Often depleted [8,10]	2	4	-		17.055
Actinomycetota	Mixed functions, some beneficial		6	0	-		17.550
Bacteroidota	Contains SCFA producers as well as pathobionts	Can be enriched [8,46] or depleted [47]	2	4	-		9.832
Pseudomonadota	Contains many pathobionts	Enriched in PD [48]	2	4	-		0.762
Desulfobacterota	H_2_S-producing bacteria	Enriched in PD [49]	1	5	-		0.206
Methanobacteriota	CH_4_-producing	Enriched in PD [9]	2	4	-		0.862
Family							
Ruminococcaceae	Contains SCFA producers	Can be depleted [50] or enriched [11]	3	3	-		7.500
Bifidobacteriaceae	Contains SCFA producers, anti-inflammatory, contains probiotic species	Often enriched [38,51]	5	1	-		12.501
Enterobacteriaceae	Gram-negative, LPS producers, implicated in neuroinflammation	Often enriched [10]	1	2	-	3	0.123
Desulfovibrionaceae	H_2_S producers	Enriched [52], linked to α-synuclein aggregation [52]	0	5	1	-	0.205
Erysipelotrichaceae	Contains SCFA producers, increased in inflammatory diseases [53] and disrupted lipid metabolism [54]	Enriched [38] or depleted [55], correlated with worsening UPDRS-III [38]	1	5	-		0.868
Genus							
SCFA Producers Reported as Reduced in PD Compared to HCs							
*Faecalibacterium*	Key SCFA producer, anti-inflammatory, supports gut barrier, reduces systemic and neuroinflammation	Depleted in PD [56,57,58]	4	1	1	-	3.019
*Anaerostipes*	SCFA producer	Depleted [9], protective against PD [59]	0	2	4	-	2.635
*Blautia*	SCFA producer	Reduced in PD [60], negatively associated with PD severity [61]	1	2	3	-	13.194
*Roseburia*_*A*	SCFA producers, anti-inflammatory, reduces systemic and neuroinflammation	Reduced in PD [60]	0	5	-	1	0.872
*Roseburia_C*			0	4	-	2	0.149
*Coprococcus_A_187866*	SCFA producers, anti-inflammatory	Reduced in PD [60]	1	2	2	1	0.275
*Coprococcus_A_121497*			0	4	0	2	0.265
SCFA Producers Reported as Increased in PD Compared to HCs							
*Bifidobacterium*	SCFA producer, enhances tight junctions [62], neuroprotective in other models	Often enriched [8], but low levels found correlated with faster progression [63]	4	0	2	-	12.498
*Alistipes*	SCFA producer, mixed roles, beneficial and detrimental (IBD) effects [64]	Often enriched [9]	2	4	-	-	1.169
*Parabacteroides*	SCFA producer, anti-inflammatory in the microbiome	Can be enriched [65]	1	4	1	-	1.486
SCFA Producers Reported as Either Reduced or Increased in PD Compared to HCs							
*Gemmiger*	SCFA producer	Sometimes enriched [66], other times depleted	1	3	2	-	3.533
*Prevotella*	Some strains related to dysbiosis, SCFA producer	Can be depleted [67] or enriched [68], inversely correlated with disease progression [34]	3	0	-	3	0.622
*Turicibacter*	SCFA producer, modifies bile acids, reduces cholesterol and triglycerides (mice)	Depleted [16] or enriched [69]	4	1		1	0.065
*Eubacterium_R*	SCFA producers, mixed species	Depleted [70] or enriched [71], some species correlated with higher UPDRS [70]	0	4	1	1	0.630
*Eubacterium_J*			1	3	-	2	0.364
*Eubacterium_G*			1	5	-	-	0.163
*Eubacterium_F*			1	1	1	3	0.083
*Eubacterium_I*			0	2	2	2	0.078
*Butyricimonas*	SCFA producers	Enriched in PD [71], higher abundance correlated with worse cognitive symptoms [72] but better non-motor symptoms in one study [45]	4	0	1	1	0.054
*Ruminococcus_B*	SCFA producers, strain-specific interactions in health and disease [73]	Can be depleted [69] or enriched in PD [42]	0	4	-	2	0.234
*Ruminococcus_E*			1	3	1	1	0.042
*Bacteroides*	SCFA producers, some pro-inflammatory strains	Enriched [74] or depleted [67] in PD, low levels correlated with faster progression [63]	2	4	-	-	5.051
Pathobionts—Reported as Enriched in PD Compared to HCs							
*Streptococcus*	Pathobiont	Enriched in PD [8]	1	0	5	-	2.381
*Limiplasma*	Unknown	Enriched in PD [9], correlated with PD severity [75]	2	2	1	1	0.375
*Collinsella*	Related to a high-protein and low-fibre diet [76], may be pro-inflammatory	Enriched in PD in some studies [36,66], depleted in one Indian study [37], related to Lewy Body dementia [77], correlated with faster PD progression [35]	5	1	-	-	3.905
*Methanobrevibacter*	Archean CH_4_ producer	Enriched in PD [9]	0	2	1	3	0.190
*Klebsiella*	LPS producer	Enriched in PD [65]	1	2	-	3	0.110
*Bilophila*	H_2_S producer	Correlated with PD progression [35]	1	3	-	2	0.059
*Desulfovibrio*	H_2_S producer	Enriched, correlated with worsened MDS-UPDRS-III and IV [38]	0	2	2	2	0.139
*Holdemania*	Associated with obesity [78]	Over-represented in PD [42]	0	6	-	-	0.054
Other Genera							
*Barnesiella*	Mixed effects, may ameliorate T2D [79]	Reduced abundance correlated with faster PD progression [80]	3	3	-		0.402
*Akkermansia*	Mucin degrader, gut barrier support [81]	Often enriched [35], may induce α-synuclein in vitro [82], neuroprotective in a mouse model of PD [83]	2	4	-	-	2.234

PD = Parkinson’s disease; HCs = healthy controls; SCFA = short-chain fatty acid; MDS-UPDRS = Movement Disorder Society Unified Parkinson’s disease Rating Scale; nd = not detected; nc = no change.

## Data Availability

The data presented in this study are openly available in NCBI website. 16S rDNA sequences are available under the NCBI BioProject accession number PRJNA790457.

## Supplementary Materials

The following supporting information can be downloaded at: https://www.mdpi.com/article/10.3390/jcm15010368/s1, Table S1: Parameters of the photobiomodulation devices and treatment used in the study. Table S2: Taxonomic changes over 5 years.
