# Supplementary material for: Five-Year Follow-Up of Photobiomodulation in Parkinson’s Disease: A Case Series Exploring Clinical Stability and Microbiome Modulation
Source: J Clin Med. 2026 Jan 4;15(1):368. doi: 10.3390/jcm15010368 (PMC12786582; doi:10.3390/jcm15010368)
Supplement: Supplementary file 1 [file jcm-15-00368-s001.zip › Supplementary Table S1.pdf]

Supplementary Table S1. Parameters of the photobiomodulation devices and treatment used in the study.

PARAMETER	SYMBYX PDCare LASER	SYMBYX NEURO		VIELIGHT NEURO GAMMA			WELLRED Duo Coronet	
Manufacturer	Spectra Analytic Irradia AB	SYMBYX Pty Ltd		Vielight Inc.			Well Red Pty Ltd	
Diodes	2 x 904nm laser diodes	40 x LED		5 x LED			80 x LED	
Wavelength	904nm	635nm + 810nm		810 nm			670nm + 810nm	
Laser class	1 (home use laser)	n/a		n/a			n/a	
				posterior	anterior	nasal		
Number of diodes	2	20 (635nm)	20 (810nm)	3	1	1	40 (670nm)	40 (810nm)
Output power	30 mW	27 mW	52 MW	100 mW	75 mW	25mW	undisclosed	undisclosed
Peak power	25,000 mW	n/a		n/a	n/a	n/a	n/a	n/a
Pulse frequency	50 Hz			40 Hz	40 Hz	40 Hz	40 Hz	40 Hz
Beam spot size	0.635 cm^2			~1 cm	~1 cm	~1 cm	undisclosed	
Power density per diode	47 mW/cm^2			100 mW $/cm^2$	75 mW $/cm^2$	25 mW $/cm^2$	362 mW $/cm^2$	234 mW $/cm^2$
Total output power	60mW	540 mW	1040 mW	400 mW			undisclosed	
Irradiation time per point	60 s			2100 s			720 s	720 s
Total irradiation time	660 s	720 s	720 s	2100 s			720 s	720 s
Total energy per point	3.6 J	19.44 J	37.44 J	60 J	45 J	15 J	undisclosed	undisclosed
Number of sites	10 (9 abdomen, 1 neck)	20	20	3	1	1	40	40
Total energy dose per treatment	39.6 J	388 J	749 J	180 J	45 J	15 J	undisclosed	undisclosed
Treatment frequency	3 x per week	3 x per week		3 x per week			3 x per week	
